# 在线样品制备技术耦合液相色谱-质谱系统在食品危害物检测中的应用进展

**DOI:** 10.3724/SP.J.1123.2023.04026

**Published:** 2023-12-08

**Authors:** Hongwen ZHAI, Hongyu MA, Meirong CAO, Mingxing ZHANG, Junmei MA, Yan ZHANG, Qiang LI

**Affiliations:** 河北省食品检验研究院,河北省食品安全重点实验室,国家市场监管重点实验室(特殊食品监管技术),特殊食品安全与健康河北省工程研究中心,河北石家庄050227; Hebei Food Safety Key Laboratory, Key Laboratory of Special Food Supervision Technology for State Market Regulation, Hebei Engineering Research Center for Special Food Safety and Health, Hebei Food Inspection and Research Institute, Shijiazhuang 050227, China

**Keywords:** 在线样品制备技术, 在线固相萃取, 管内固相微萃取, 湍流色谱法, 食品危害物, 综述, on-line sample preparation techniques, on-line solid phase extraction (on-line SPE), in-tube solid phase microextraction (in-tube SPME), turbulent flow chromatography (TFC), food hazards, review

## Abstract

食品安全检测具有重要意义,但食品样品基质复杂,测定其中危害物时,通常需要以下几个步骤:样品制备,即采用适合的样品前处理方法,在不同基质中将目标物分离出来;分离纯化,利用色谱系统进一步分离纯化目标物;定性定量分析,基于目标化合物的性质选择合适的检测器进行分析。其中样品制备是关键步骤之一,将样品制备过程与液相色谱系统耦合可以实现样品的在线自动化分析。与传统人工处理过程相比,在线分析不仅能够减少人工操作误差,保证良好的精密度和重复性,而且可以降低溶剂消耗,避免样品间交叉污染,同时节约分析时间,提高检测效率。本文主要介绍目前常用的在线样品制备技术,包括在线固相萃取(on-line SPE)、管内固相微萃取(in-tube SPME)、湍流色谱法(TFC),详细阐述了其基本原理和耦合设备。在线样品制备技术耦合液相色谱-质谱系统分为两个维度,主要依赖于阀切换技术,将样品制备(第一维度)和液相色谱系统(第二维度)之间建立物理连接,随后采用相应的检测器进行分析。第一维度的作用主要是去除样品杂质,净化分离目标物,为第二维度对目标物进行定性定量检测做准备。此外,文章对3种在线净化系统适用的不同净化填料进行了总结讨论。最后综述了近10年来3类在线系统在食品兽药残留、农药残留、毒素污染以及其他危害物检测中的应用和研究进展,并对该领域存在的问题和发展趋势进行了探讨和展望,以促进在线样品制备技术进一步应用于食品安全检测。

## Abstract (en)

Food safety has received increased attention, and food detection is of great significance. The food matrix is complex, and diverse food hazards have been identified. Thus, the detection methods and sample preparation techniques for food matrices must be continuously optimized and updated. Several steps are usually required when a chromatographic system is used to determine food hazards: sample preparation, that is, the separation of targets from different substrates using a suitable preprocessing method and target-substance separation and purification, which is usually achieved using chromatographic separation. The selection of an appropriate detector for qualitative and quantitative analyses is usually based on the properties of the target compound. The sample preparation procedure is considered the most time-consuming aspect of the entire food-analysis process. It is also prone to analytical errors. Therefore, optimization of the sample preparation process is a key issue in the field of chemical analysis. Researchers have developed a series of new, efficient, and accurate sample preprocessing methods, and an on-line sample-preparation system has been found to be a feasible approach. On-line sample preparation coupled with liquid chromatography-mass spectrometry (LC-MS) presents many advantages. First, manual operation could reduce analytical errors to ensure good accuracy and repeatability. It could also reduce the consumption of chemical reagents and avoid cross-contamination between samples. Furthermore, an on-line sample-preparation system could shorten the sample-preparation time and improve the detection efficiency. On-line sample preparation coupled with LC-MS has been widely applied in the fields of environment, biology, and food. On-line sample preparation systems coupled with LC-MS are divided into two modules: the first modules involves sample preparation and the second module involves the LC system. The first module remove impurities and isolates the target compounds in preparation for their qualitative and quantitative detection. The coupling of these two modules depends mainly on valve switching. In this paper, we introduce the most frequently used on-line sample-preparation techniques, including on-line solid phase extraction (on-line SPE), in-tube solid phase microextraction (in-tube SPME), and turbulent chromatography (TFC). We then describe the basic principles and coupling equipment of these three on-line analytical technologies in detail. The coupling equipment establishes a physical connection between the two modules. Next, we discuss the properties of different purification fillers in an on-line sample-preparation column. The applications and research progress of on-line systems for pesticide residues, veterinary drug residues, and biotoxins are also discussed. Compared with offline sample preparation, on-line analytical systems present several advantages. On-line analytical systems can not only greatly reduce the analysis time and solvent consumption but also improve the detection sensitivity and accuracy. Such systems can be used to determine food hazards to ensure food safety. Finally, the existing problems and development trends of on-line analytical systems are discussed and prospected. To promote the applications of on-line analytical technology in food-safety detection, we suggest that the following three aspects be considered. First, more on-line purification columns with novel fillers, in addition to C18 or polymer fillers, should be developed. Second, compared with ordinary detectors, high-resolution MS detectors have better precision and accuracy. Coupling on-line analytical technologies with a high-resolution mass spectrometer may be beneficial for the further development of on-line analyses. Third, different food matrices should be compared and evaluated to continuously optimize the detection process and improve the efficiency of on-line analytical systems. As concerns regarding food safety issues have increased, the applications of on-line analytical technologies for food detection can be expected to become increasingly important.

在常规化学分析过程中,样品制备是最耗时、最费力的步骤,占据了整个过程2/3的时间,同时超过1/3的分析误差是在样品制备过程中产生的^[1]^。优化样品制备程序、缩短样品制备时间,同时减少分析误差、提高检测精度是化学分析领域发展的关键问题之一。样品制备过程需要考虑多个因素,包括人力、时间、耗材等经济成本以及方法准确性和适用性。传统的样品制备过程多为离线方法,需人工操作,耗时长、误差大且重复性差^[2]^。在线样品制备技术是近年来发展的一种前处理手段,其将样品制备过程与液相色谱系统在线耦合,自动化处理样品,实现目标物的在线净化和富集,具有以下优点:(1)利用在线设备处理样品,减少人为操作误差,精密度和准确性高;(2)与离线方法相比,样品制备时间短,溶剂消耗少;(3)样品制备过程在封闭系统内进行,有效避免样品污染以及目标物的降解^[3⇓-5]^。

20世纪80年代,Barceló等^[6]^将SPE和液相色谱系统结合测定环境水体中痕量农药及其降解产物,为在线分析提供了思路。此后,在线样品制备技术广泛应用于环境、生物以及食品样品检测领域^[7⇓⇓⇓-11]^。我们实验室也对在线样品制备技术进行了一些研究,建立了一系列准确高效的在线分析方法,包括将SPE与二维液相色谱仪在线耦合测定特医食品中营养元素维生素A、D、E^[12]^;在线SPE-HPLC-MS/MS测定玉米粉和小麦粉中的伏马毒素和脱氧雪腐镰刀菌烯醇等毒素^[13,14]^;在线SPE-HPLC-MS/MS测定猪肉和羊肉中的10种*β*-受体激动剂^[15]^;湍流色谱法(TFC)耦合HPLC-MS/MS测定白菜和黄瓜中的15种农药残留^[16]^。在分析食品基质中各类危害物时,图1是一个典型的分析工作流程。首先是样品制备,可以采用在线(主要有在线固相萃取(on-line SPE)、管内固相微萃取(in-tube SPME)、TFC等)和离线(包括液液萃取(LLE)、固相萃取(SPE)、固相微萃取(SPME)、搅拌棒吸附萃取(SBSE)和填充吸附微萃取(MEPS)等)两种方式^[17]^;样品经前处理后,利用色谱系统(气相色谱(GC)、液相色谱(LC)、超临界流体色谱(SFC)和多维色谱(MDC)等)将目标化合物进一步分离;最后进入不同检测器进行定性定量分析。在离线模式下,两个设备之间没有物理连接,样品溶液需要手动传输到色谱系统^[17]^。而在线模式,则将样品制备过程(作为第一个维度)和色谱分离(作为第二个维度)之间建立物理耦合,样品直接从制备步骤流向色谱分离步骤,实现自动化传输^[18,19]^。在线样品制备设备主要是基于阀切换技术,通常利用六通阀或十通阀,将目标物自第一维度转移至第二维度。其中,针对SPE、SPME、TFC与液相色谱系统耦合的研究较多,应用较广。本文主要对这三类技术进行综述,详细介绍其原理、仪器装置特点及相似之处,最后讨论其在食品分析领域的主要应用进展。

**图1:**
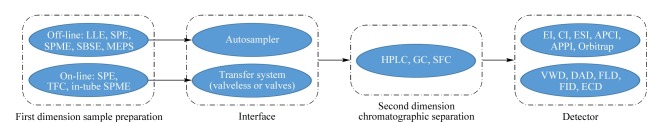
典型在线样品制备技术耦合色谱技术的工作流程图

## 1 在线样品制备技术

### 1.1 在线固相萃取技术

固相萃取技术与色谱分离原理类似,所以其与液相色谱设备在线联用易实现在线多维分离^[20]^。与离线固相萃取相比,在线固相萃取更具有优势,如自动化程度高、样品处理步骤简单等^[21,22]^。

如图2所示,SPE-LC在线联用装置是较为简单的多维分离设备,不仅可用于在线固相萃取,进行微小调整后,也可以用于其他样品制备技术,如In-tube SPME和TFC。该系统主要由六通阀(或十通阀)、加载泵、洗脱泵、SPE柱(第一维)、色谱分析柱(第二维)组成。在线SPE的工作步骤为平衡、上样、淋洗、洗脱和洗涤。具体工作流程为(1)平衡:六通阀位于加载(Load)位置,由加载泵载入流动相冲洗SPE柱,达到平衡状态,为接下来的步骤做准备;(2)上样:六通阀位于Load位置,自动进样器将样品注入SPE柱;(3)淋洗:六通阀位于Load位置,加载泵不断淋洗流动相带走杂质化合物,SPE柱对目标物富集净化,此步需要优化淋洗时间,太短不能充分去除杂质化合物,太长则有可能导致目标物损失;(4)洗脱:六通阀切换到进样(Inject)位置,由洗脱泵将目标物洗脱下来,转入第二维进行分析检测;(5)洗涤:六通阀切换到Load位置,利用高比例有机相进一步去除残留在SPE柱上的杂质化合物,清洗完成后流动相回到初始比例,等待下次进样,并重复以上步骤^[23,24]^。在线SPE过程一般使用低于5 mL/min的流速和合适的洗脱溶剂,避免峰展宽、峰拖尾等现象。

**图2:**
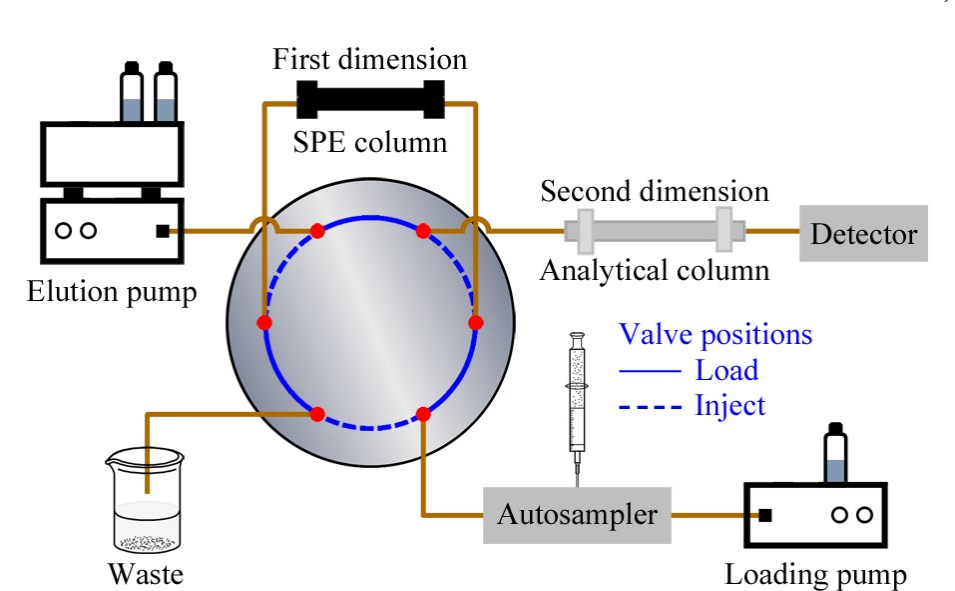
在线SPE-LC装置示意图

SPE-LC在线联用装置洗脱过程根据洗脱溶剂与萃取溶剂流动方向分为正向冲洗和反向冲洗。图2所选用的反冲模式在实际工作中应用较为广泛,SPE柱头部高度保留的目标物通过反向洗脱充分转移至分析色谱柱,最大限度地减少峰展宽效应。但采用反向冲洗模式,特别是进样量较大(大于100 μL)时,容易将杂质带入分析柱,造成堵塞,因此样品提取后,需要适当的过滤或者离心后进入在线SPE装置^[24]^。

在实际样品检测中,目标分析物含量低,基质效应影响大,在线SPE柱填料的选择尤为重要。在线SPE柱填料通常可分为4种类型:键合硅胶型填料、高分子聚合物型填料、吸附型填料、混合型填料及专用填料。硅胶基质柱主要用于弱极性和中性目标物的分析,适用范围广,应用广泛,但是pH耐受范围窄^[25]^。聚合物基质填料对极性和中性大分子物质分离性能良好,且使用pH范围宽、化学稳定性高,但是针对性差,选择性低,共提取样品溶液杂质成分多,给二维分析检测带来不便^[26]^。离子交换吸附剂主要适用于带电荷的目标物质。分子印迹聚合材料和免疫亲和吸附剂可以特异性识别目标物,具有良好的选择性,从而大大提高二维分析柱的分析效率^[27]^。目前Waters公司开发了不同填料的商用在线SPE柱,包括C18柱、HLB柱、MCX柱、MAX柱、WCX柱和WAX柱,为在线制备技术的研究提供了便捷。

各类目标分析物千差万别,建立在线样品制备方法要根据其性质选择相适应的在线净化柱。为促进在线分析方法在食品检测领域的应用,研究人员针对不同物质特性开发制备出了各种新型净化柱填料。Nian等^[28]^开发校偶介孔聚合物(scholl-coupling mesoporous polymer, NH_2_@SMPA)作为在线固相萃取填料分析豆芽中6种植物生长调节剂。Lu等^[29]^合成了溴化共价有机骨架(brominated covalent organic framework, Br-COF)作为在线净化柱填料,将在线SPE与液相色谱耦合测定食品中6种多溴联苯醚,Br-COF与商业吸附剂相比,萃取效果更好,特异性更强。Sun等^[30]^采用SPE-UPLC-MS/MS测定海产品中痕量全氟和多氟烷基物质,制备了对全氟和多氟烷基物质具有快速吸附平衡和高吸附量的离子共价有机骨架(ionic covalent organic framework, TPB-BFBIm-iCOF)填料。分子印记材料Polymyxin E能够特异性地保留与其分子大小和空间结构相似的多肽类抗生素, Song等^[11]^将其作为填料制备在线固相萃取柱,有效富集和纯化动物组织中的多肽类抗生素。

### 1.2 在线管内固相微萃取技术

In-tube SPME是一种微型样品制备技术,在细管或者毛细管内涂有固定相,样品共提取液通过毛细管时目标物质被保留,杂质成分被去除。在线In-tube SPME系统萃取目标物的方式有两种:一种是样品溶液以一个方向通过毛细管系统进行萃取;另外一种是在毛细管内重复抽吸样品溶液进行萃取^[31,32]^。目标物解吸后被转移至色谱分析柱进行分离和测定,解吸分为静态和动态两种方式^[33]^。

流动萃取模式需配置一个自动阀,类似于在线SPE装置(图2)。在In-tube SPME装置中,毛细管(第一维度)连接在六通阀相应的端口上,加载泵将样品溶液经自动进样器载入毛细管内,洗脱泵载入洗脱溶剂,并将被提取的目标物质洗脱至二维分析柱以分离检测。利用阀门的切换来完成在线分析的所有步骤:当阀门处于加载位置时,样品溶液进入到毛细管内,同时目标分析物被固定到毛细管内;阀门切换至进样位置时,洗脱溶液进入毛细管内对目标物质进行洗脱^[34]^。

重复抽提模式(图3)需要重新对自动进样器进行配置,将毛细管连接于进样针和样品环之间。这种模式下,六通阀和计量泵包含于自动进样器模块之中,计量泵用于在进样瓶中循环吸取样品,直至完成提取过程。但是,这种模式需要匹配相应的软件,能够自动控制不断抽取样品。如图3,当六通阀保持在加载位置时,洗脱泵直接连接至二维分析柱。此时,样品被重复抽取,直至目标物提取完全,这个过程需要持续较长时间。因此,为提高目标物的回收率,必须优化循环提取的次数。洗脱步骤,六通阀切换到进样位置,流动相通过毛细管,将提取的目标物解吸下来,转移至分析柱进行定量分析^[35]^。

**图3:**
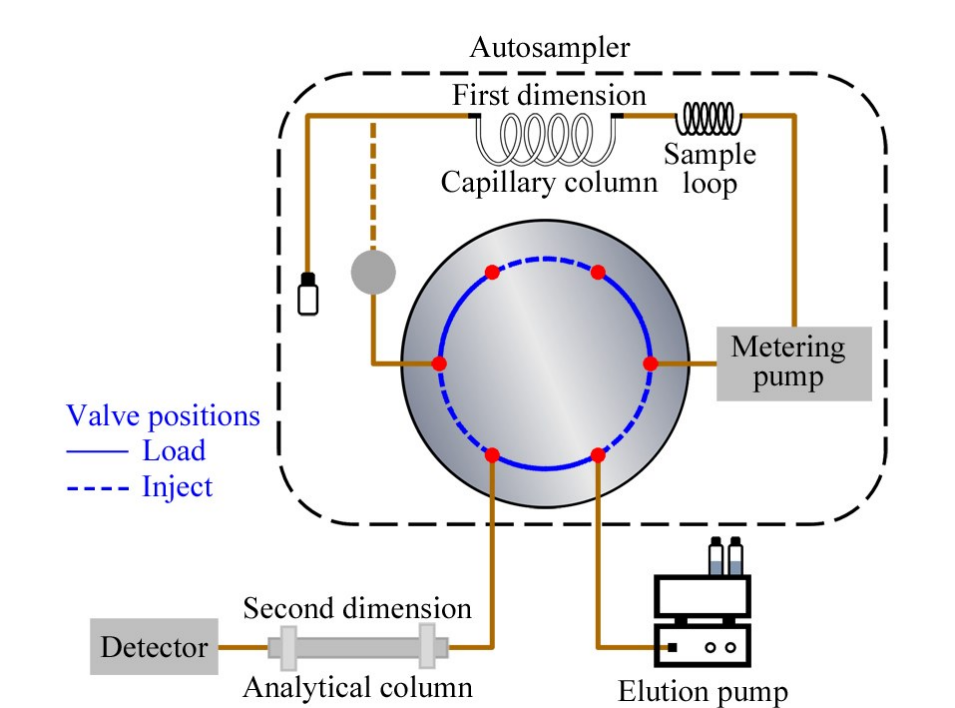
在线In-tube SPME-LC装置示意图

毛细管内径较小(大多小于1 mm,研究中常用的有0.32、0.50和0.53 mm等)导致系统压力较高,增加了重复提取的难度。不同类型的萃取相被用于In-tube SPME,特别是壁涂开口管柱(WCOT柱),其萃取相以薄膜的形式涂在毛细管壁上,可以提供更好的渗透性,在实际分析中被普遍应用^[36]^。

In-tube SPME萃取体系的效率和选择性取决于萃取速率、样品体积、样品pH值、固定相类型以及毛细管柱内径、长度和膜厚等因素。选择性材料被用于制备毛细管,除了聚二甲基硅氧烷等传统涂料外,还引入了许多新的In-tube SPME涂料,如单壁碳纳米管、聚合物、单片材料等^[32]^。将In-tube SPME与LC在线耦合的优势有很多,如小型化和自动化能力、高通量性能以及低溶剂消耗^[36]^。

### 1.3 湍流色谱法在线净化技术

TFC是在20世纪90年代中期发展起来的一种分析工具,它是基于流体在高流速下表现出来的非流体特征,将样品中的大分子(如蛋白质、色素、药物)与小分子分析物分离开来^[37]^。TFC在线净化技术是近年来发展起来的自动在线样品制备技术,与液相色谱结合,将复杂样品直接注入仪器分析系统中。

TFC在线净化技术通常使用粒径大且不规则的颗粒作为填料,是一种将涡流扩散、化学作用以及体积排阻的原理相结合的样品前处理技术^[38]^。TFC柱可采用不同的萃取相,表面修饰不同化学基团使其具有特殊的吸附性,主要有3种类型:(1)硅胶基质柱(如C8、C18、Cyclone C2、Cyclone Fluro)对非极性物质保留性较强;(2)离子交换柱(如MCX、MAX、WCX、WAX)适用于酸性(如磺酸盐类)或碱性(如季铵盐类)化合物;(3)聚合物基质柱(如HLB、Cyclone、Cyclone-P)可用于分析极性目标物。Thermo Scientific公司特有大孔填料TFC柱,包括保留极性和非极性分析物的HyperSep Retain PEP,保留碱性和非极性分析物的HyperSep Retain CX,保留酸性和非极性分析物的HyperSep Retain AX和保留高极性分析物的HyperSep Hypercarb等。在线TFC柱平均颗粒直径为30~60 μm。最初,TFC色谱柱被设计为内径1 mm,流速大于4 mL/min才能达到涡流条件,随着柱径从1 mm减小到0.5 mm,溶剂消耗减少,且流速大于1.5 mL/min就能达到湍流流动条件^[39]^。高流速产生的涡流增加了流动相内的传质作用,大分子物质传质作用差,扩散效率低,小分子化合物可以进入到填料表面的孔隙中,因此,TFC在线净化柱能够保留小分子,而排除蛋白质等大分子物质^[40]^。

TFC-LC耦合方式有多种,其中聚焦模式是最常用的(图4)。聚焦模式系统适用于复杂基质中多残留分析,有良好的灵敏度和重现性。聚焦模式是完全自动化的系统,需要2个六通阀(V1和V2)和2个色谱泵(四元加载泵和二元洗脱泵)来实现不同流路的自动切换,同时六通阀V2仅需4个端口,剩余2个未使用的端口安装盲塞。样品加载、转移、洗脱和调节(再平衡)是TFC在线系统的主要分析步骤。样品加载时,阀门V1和V2处于Load位置,样品自进样器随高流速流动相进入到TFC柱(第一维),小分子分析物被提取出来,固定在TFC柱中,大分子杂质则被排除,流入废液;接下来,阀门V1和V2切换到Inject位置,流路中的洗脱溶剂通过TFC柱将目标分析物解吸出来,并转移至分析柱(第二维),在这一过程中,进样泵的流速降低,从而将分析物富集在分析柱的柱头上;此后,阀门V2重新转到Load位置,分析柱对目标进行进一步分离检测,同时冲洗TFC柱,避免遗留效应和交叉污染^[41,42]^。阀门V1保持在Inject位置,使回路充满解吸溶剂。最后,2个阀门回到初始位置(Load位置),平衡TFC柱,为分析下一个样品做准备。

**图4:**
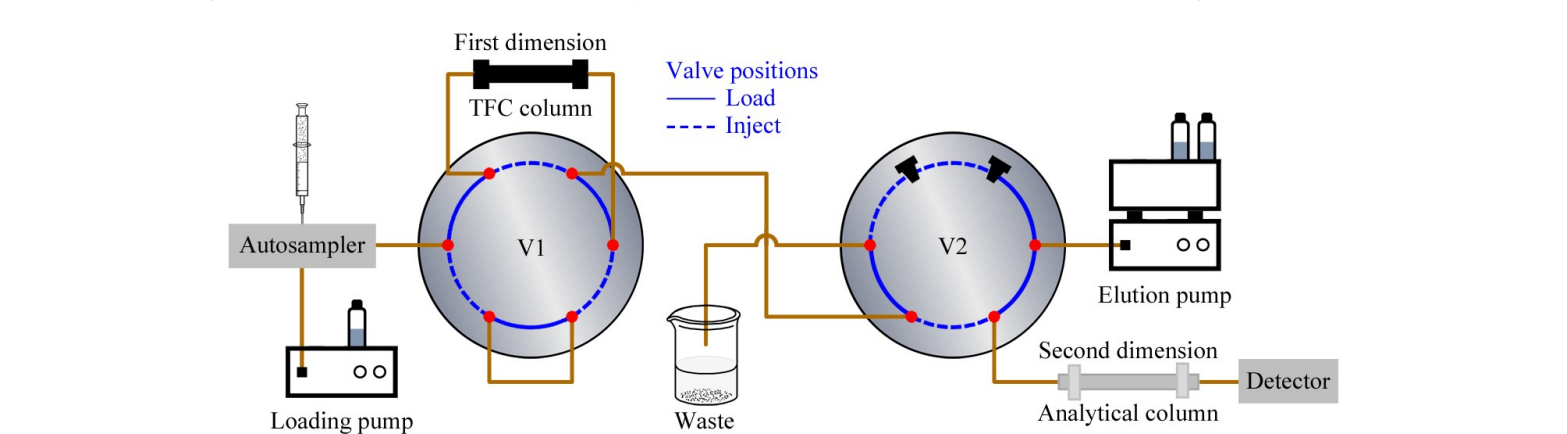
在线TFC-LC装置示意图

Franco等^[43]^提出了一种基于聚焦模式下的TFC在线净化新装置,仅使用一个自动化的十通阀来替代上述2个六通阀,该系统其他元件与上述相同,额外增加了一个盲塞和一个三通接头,将来自加载泵和洗脱泵的流动相与分析柱连接起来。但是,在这种设置中,TFC柱只能进行正向冲洗(与提取液流动方向一致),而在前面描述的2个阀门设置中,既可进行正向冲洗又可进行反向冲洗。因此,这种装置可能需要更长的清洗时间,以确保TFC柱中的基质干扰化合物完整、有效地去除。

在线SPE结构配置也可以用于TFC系统,但是需将SPE柱替换为TFC柱,或者使用可以适应高流速的SPE柱。在线SPE结构配置相对简单,但存在局限性,不能有效地将分析物聚焦于分析柱的头部,二维分析柱分离目标物时,导致峰形变宽,分离效率降低,不利于多种化合物的分析^[42]^。

## 2 在线样品制备技术耦合液相色谱-质谱系统在食品检测中的应用

农药、兽药在促进农业、畜牧业生产方面做出了巨大贡献,但残留在食品中的药物对人体健康会产生多方面不利影响,如变态反应、过敏反应、细菌耐药性等。此外,食品中的生物毒素因毒性强、污染面广,也成为食品安全检测中重点关注的对象。因此,政府机构规定了食品中有害物质的最大残留限量(maximum residue limit, MRL)以确保食品安全,MRL通常对应非常低的分析物浓度,需要更加先进的样品制备技术来满足实际检测需求^[7]^。离线样品制备一般需要提取和净化,耗费人力和时间,而采用在线样品制备技术,样品在经简单的提取(甲醇、乙腈等溶剂)后,即可使用仪器自动化分析,省去了样品净化过程,更加高效、准确,在食品检测中应用广泛。

### 2.1 食品中兽药残留检测

在线样品制备技术应用于食品中各类兽药残留的检测,如磺胺类、大环内酯类、喹诺酮类抗生素,表1列举了3种在线联用设备在兽药残留检测方面的应用。针对磺胺类抗生素,Li等^[44]^对其在鱼类中的在线分析方法进行了研究,发现在样品预处理过程中使用单一在线柱,如HLB或C18,无法从样品中去除可能引起基质效应的磷脂,创新性地将混合阳离子交换柱(Oasis^®^ MCX)与亲水亲脂平衡柱(Oasis^®^ HLB)串联用于在线固相萃取净化,以消除基质干扰,其中Oasis^®^ MCX柱用于纯化,Oasis^®^ HLB柱用于Oasis^®^ MCX柱与色谱柱之间的转换,这种在线固相萃取结合UHPLC-MS/MS的方法为复杂基质中抗生素的自动化、高效率检测指出了新的方向。此研究对实际样品所有目标物检出的准确度为80%~101%,与传统方法相比,检出限(1.46~15.5 ng/kg)和定量限(49~51.6 ng/kg)更低,准确度更高。顾欣等^[51]^建立了在线SPE-LC-MS/MS检测牛奶中14种磺胺药物残留的方法,有效避免了牛奶中含有的蛋白质和乳脂对仪器的干扰和污染,方法的检出限为0.05 μg/kg,定量限为0.1 μg/kg,回收率在60%~90%范围内,批内和批间相对标准偏差均小于10%,比离线固相萃取净化方法成本低,效率高,准确性好。除了在线SPE外,TFC和In-tube SPME也被用于食品中兽药残留的检测。Zheng等^[50]^将管内固相微萃取与液相色谱-电喷雾电离-四极杆飞行时间质谱(LC/ESI-QTOF-MS)相结合,建立了测定牛奶、鸡蛋、鸡肉和鱼肉中7种微量喹诺酮类抗生素的方法。样品制备过程采用在线聚甲基丙烯酸-乙二醇二甲基丙烯酸酯整体柱萃取,然后采用LC/ESI-QTOF-MS识别目标化合物,7种喹诺酮类抗生素在鸡蛋、牛奶、鸡肉和鱼肉中的检出限分别为0.3~1.2、0.2~3.0、0.2~0.7和0.2~1.0 ng/g,回收率在80.2%~115.0%范围内,RSD<14.5%,该方法检出限低,精密度和灵敏度较高。高分辨质谱的使用,进一步提高了检测精确度和灵敏度,也成为在线样品制备技术发展的新趋势。Bousova等^[47]^建立了7类(氨基糖苷类、大环内酯类、林可胺类、磺胺类、四环素类、喹诺酮类和甲氧苄氨嘧啶类)36种抗生素的鉴别和定量方法,可用于鸡肉中抗生素的检测。样品利用乙腈-2%三氯乙酸(45∶55, v/v)涡旋提取5 min,即可将提取液注入TFC在线系统,自动化除去大分子和小分子杂质,根据2002/657/EC对方法进行验证,均符合要求,与传统的净化技术相比,该方法简单、快捷、高效。

**表1:** 在线样品制备技术耦合液相色谱-质谱在食品兽药残留检测中的应用

Analytes	Matrices	First dimension	Second dimension	On-line analysis system	LODs/ (ng/kg)	LOQs/ (ng/kg)	Recov- eries/%	RSDs/ %	Ref.
10 *β*-agonists	pork and lamb	MCX column (10 mm×1 mm)	XBridge C18 column (150 mm×4.6 mm, 5 μm)	SPE-UHPLC- MS/MS	4-40	20-200	76.5- 107.7	2.4- 9.5	[15]
18 sulfonamide antibiotics	aquaculture	Oasis^®^ MCX column (20 mm×2.1 mm, 2.5 μm) and Oasis^®^ HLB column (20 mm ×2.1 mm, 2.5 μm)	F5 column (150 mm× 3.0 mm, 2.6 μm)	SPE-UHPLC-MS/MS	1.46-15.5	49-51.6	71.5- 102.0	3.47- 14.2	[44]
10 macrolide antibiotics	pork	HLB column (10 mm×1 mm)	XBridge C18 column (100 mm×2.1 mm mm, 2.5 μm)	SPE-UHPLC-MS/MS	50-300	100-1000	69.9- 115.2	0.9- 9.2	[45]
Abamectin and ivermectin	milk	SB-C18 column (30 mm×2.1 mm, 3.5 μm)	SB-C18 column (75 mm×2.1 mm, 3.5 μm)	SPE-UHPLC- MS/MS	0.65-0.67	2.21-2.23	82-88	4.8- 9.7	[46]
36 antibiotics	chicken meat	TurboFlow Cyclone- P (50 mm×0.5 mm, 60 μm)	Betasil phenyl hexyl column (50 mm×2.1 mm, 3 μm)	TFC-HPLC- MS/MS	300-40000	1000-120000	80-120	3-28	[47]
Ractopamine	beef	TurboFlow Cyclone- P (50 mm×0.5 mm, 60 μm)	Accucore C18 column (50 mm×3 mm, 2.6 μm)	TFC-HPLC- MS/MS	-	0.3	85-104	2.6- 14.0	[48]
5 benzimidazoles and 7 metabolites	milk	ADS column (25 mm ×4 mm, 25 μm)	Zorbax Eclipse XDB- C18 column (150 mm ×4.60 mm, 3 μm)	SPE-UHPLC- MS/MS	0.1-0.8	-	88-114	0.8- 9.7	[49]
8 quinolone antibacterials	milk, egg, chicken and fish	PEEK tube (poly (MAA-co-EGDMA), 150 mm×0.50 mm)	C18 column (250 mm ×2.0 mm, 5 μm)	In-tube SPME- LC/ESI-Q- TOF-MS	0.3-1.2 (egg), 0.2-3.0 (milk), 0.2-0.7 (chick- en), 0.2-1.0 (fish)	1.0-4.0 (egg), 0.6-10 (milk), 0.8-2.4 (chick- en), 0.6-3.3 (fish)	80.2- 115.0	1.3- 14.5	[50]

### 2.2 食品中农药残留检测

在水果、蔬菜以及粮食作物的生产加工等过程中,农药的使用越来越频繁,农药残留问题日益严重。因此,针对农药残留问题,研究人员不断开发新方法,采用新手段,在线分析方法也不断被应用于农残检测(表2)。Pérez-Mayán等^[52]^将基于反相聚苯乙烯-二乙烯苯吸附剂的SPE柱与LC-MS/MS在线耦合,自动监测葡萄酒中37种杀菌剂和11种杀虫剂。通过优化各项参数,如进样体积、洗脱条件等,48种农药的定量限均低于2.5 μg/L,并且回收率和重复性良好,为葡萄酒中杀菌剂和杀虫剂的检测提供了新的解决方法。张海超等^[53,54]^对采用在线净化技术检测农药残留物质进行了多项研究。一是将TFC在线净化技术与四极杆轨道阱高分辨质谱(Q-Orbitrap HRMS)相结合,可在无标准物质的情况下完成果蔬中212种农残的高通量筛查和确证,定量限均可达到5 μg/kg,准确性和灵敏度良好,方法采用的TFC在线前处理方法不仅简化了样品制备过程,而且有效避免了传统净化方式中固相萃取填料石墨化炭黑及PSA对含平面结构农药或酸性农药的吸附^[53]^;二是采用poly(BMA-co-EDMA)整体柱作为第一维度,对大米中15种酰胺类除草剂进行在线净化提取后,耦合LC-MS/MS进行定性定量分析,该方法定量限为0.5~5.0 μg/kg,回收率为75.5%~121.3%,精密度均小于15%。实验表明TFC在线净化方法可以有效去除大米中的淀粉和油脂,且整体柱性质稳定,兼容性良好,可以反复使用,节约成本^[54]^。

**表2:** 在线样品制备技术耦合液相色谱-质谱在食品农药残留检测中的应用

Analytes	Matrices	First dimension	Second dimension	On-line analysis system	LODs	LOQs	Recov- eries/%	RSDs/ %	Ref.
48 pesticide residues	wine	PS-DVB column (12.5 mm×2.1 mm, 15-20 μm)	Zorbax Eclipse XDB- C18 column (100 mm ×2.1 mm, 3.5 μm)	SPE-HPLC- MS/MS	-	0.02-2.5 μg/L	70-119	-	[52]
212 pesticides	fruits and vegetables	TurboFlow Cyclone-P (50 mm×0.5 mm, 60 μm)	Accucore aQ C18 col- umn (150 mm×2.1 mm, 2. 6 μm)	TFC-LC/ESI- Q-Orbitrap	-	0.5-5 μg/kg	58.3- 129.4	2.8- 16.0	[53]
15 amide herbicides	rice	poly(BMA-co-ED- MA) column (50 mm× 2.1 mm)	Hypersil GOLD col- umn (150 mm×2.1 mm, 5 μm)	TFC-HPLC- MS/MS	0.20-2.0 μg/kg	0.50-5.0 μg/kg	75.5- 121.3	2.89- 12.38	[54]
107 pesticides and metabolites	raw and drinking water	XBridge C18 column (30 mm×2.1 mm, 10 μm)	ACQUITY UHPLC HSS T3 column (100 mm× 2.1 mm, 1.8 μm)	SPE-UHPLC- MS/MS	0.03-1.5 ng/L	0.1-5.0 ng/L	61.2- 119.8	0.3-18.6	[55]
15 pesticide residues	cabbage and cucumber	XL C18 (50 mm×0.5 mm, silica type)	Phenomenex C18 col- umn (100 mm×2.1 mm, 1.7 μm)	TFC-HPLC- MS/MS	0.2-1.0 μg/kg	0.5-2.0 μg/kg	75.3- 103.7	0.3- 9.5	[16]
8 carbamate pesticide residues	tomato, rice, and cabbage	CAPCELL PAK C18 column (50 mm×2 mm, 15 μm)	ACQUITY UHPLC CSH C18 column (100 mm×2.1 mm, 1.7 μm)	SPE-HPLC- MS/MS	0.01-0.3 ng/mL	0.05-1.0 ng/mL	73.76- 112.32	1.28- 13.14	[56]

### 2.3 食品中生物毒素的检测

生物毒素主要包括真菌毒素和海洋生物毒素,毒素污染影响食品安全,因此需要不断发展检测技术,加强检测水平^[57]^。表3总结了不同基质中各类毒素的在线测定方法,可以看出在线净化技术在毒素检测领域也起到重要作用。Xu等^[58]^利用在线SPE对鱼类样品进行净化,优化预处理条件(SPE柱类型、洗脱溶剂以及阀切换时间等),建立了在线SPE-UHPLC-MS/MS同时测定鱼类中8种常见微囊藻毒素的方法,方法验证实验表明,该方法基质效应干扰较弱,为0.88~1.09,加标回收率为70.5%~98.9%, RSD为1.3%~3.5%,符合美国分析化学家协会(AOAC)《标准方法性能要求指南》的精密度和准确度要求。此外,本方法微囊藻毒素-LR和微囊藻毒素-RR检出限为0.1 μg/kg,约为GB 5009.273-2016(检出限0.3 μg/kg)的1/3,证明方法可行性良好。

**表3:** 在线样品制备技术耦合液相色谱-质谱在食品毒素检测中的应用

Analytes	Matrices	First dimension	Second dimension	On-line analysis system	LODs	LOQs	Recov- eries/%	RSDs/ %	Ref.
8 microcystins	fish	XBridge C18 Direct Connect HP column (30 mm×2.1 mm, 10 μm)	Waters Cortecs C18 column (100 mm×2.1 mm, 2.7 μm)	SPE-UHPLC- MS/MS	0.1-0.5 μg/kg	0.3-1.5 μg/kg	70.5- 98.9	1.3- 3.5	[58]
Ochratoxin A	red and white wine	Oasis MAX (20 mm× 2.1 mm, 12 μm)	Chromolith^®^ FastGra- dient column (50 mm ×2 mm)	SPE-UHPLC- MS/MS	0.012- 0.014 ng/mL	0.041- 0.045 ng/mL	80-88	3-6	[59]
Fumonisins	corn and wheat flour	WAX column (10 mm ×1 mm)	XBridge C18 column (150 mm×4.6 mm, 5 μm)	SPE-HPLC- MS/MS	0.08 μg/kg	0.2 μg/kg	93.9- 117.8	1.8- 9.4	[16]
Ochratoxin A	wine	PEEK tube (150 mm× 0.50 mm, C18)	XB-C18 (100 mm×2.1 mm, 2.6 μm)	In-tube SPME-HPLC- MS/MS	0.02 μg/L	0.05 μg/L	61.4- 72.8	1.15- 6.05	[60]
Aflatoxins (B1, B2, G1, and G2)	nuts, cereals, dried fruits, and spices	PEEK tube (60 cm× 0.32 mm, Supel- Q PLOT)	Zorbax Eclipse XDB- C8 column (150 mm× 4.6 mm, 5 μm)	In-tube SPME-UH- PLC-MS/MS	2.1-2.8 pg/mL	-	80.8- 109.1	1.9- 7.7	[61]
Aflatoxin M1	milk and dairy products	BioBasic C18 (20 mm ×2.0 mm, 5 μm)	Kinetex PFP column (100 mm×2.1 mm, 2.6 μm)	SPE-UHPLC-MS/MS	0.5-0.7 ng/kg	1.5-2.4 ng/kg	86-102	3-8	[62]
13 cyanotoxins	freshwater	ZIC-HILIC column (20 mm×2.1 mm, 5.0 μm)	Poroshell 120 HILIC-Z column (50 mm×2.1 mm, 1.9 μm)	SPE-HPLC- MS/MS	0.7-4.1 ng/L	1.8-8.1 ng/L	-	1.65- 13.02	[63]
Patulin	fruit juice and dried fruit	PEEK tube (60 cm× 0.32 mm, carbox- en 1006 PLOT)	Synergi MAX-RP 80A column (150 mm×4.6 mm, 4 μm)	In-tube SPME-UH- PLC-MS/MS	23.5 pg/mL		92.5- 94.4	0.95- 4.5	[64]

赭曲霉毒素A(OTA)是污染葡萄及其深加工产品的主要真菌毒素之一,为了准确测定其在葡萄酒中的含量,Campone等^[59]^和Andrade等^[60]^分别基于在线SPE和In-tube SPME在线样品净化方法,为OTA的自动富集、纯化以及测定提供新方法。Campone等^[59]^利用在线SPE-HPLC-MS/MS方法,将OTA浓缩于Oasis MAX SPE柱上,洗涤去除干扰基质后,以反冲模式将目标物洗脱下来,在整体柱上实现了色谱分离。该方法无显著基质效应(95%~108%),检出限(0.012~0.014 ng/mL)和定量限(0.041~0.045 ng/mL)远低于欧盟委员会规定的限值;回收率和重复性符合欧盟法规No 519/2014。与传统方法相比,该方法显示出更好的分析性能,而且能够实现高通量检测和分析程序的完全自动化。Andrade等^[60]^则将In-tube SPME与HPLC-MS/MS耦合,其将C18填充的PEEK毛细管作为萃取柱,优化萃取溶剂乙腈的比例和样品加载的时间来提高在线净化效率,该方法灵敏度和精密度良好,检出限为0.02 μg/L,在巴西农业、牲畜和食品供应部(MAPA)的标准范围内。

黄曲霉毒素(AFB1、AFB2、AFG1、AFG2)主要污染各种农产品,而黄曲霉毒素M1(AFM1)是AFB1的羟基化代谢产物,是鲜牛奶中最常见的一种污染物。Nonaka等^[61]^采用In-tube SPME-HPLC-MS/MS技术,建立了一种简便、灵敏的自动测定坚果、谷物、干果和香料中AFB1、AFB2、AFG1和AFG2的方法,该方法采用Supel-Q PLOT毛细管柱作为第一维净化柱,Zorbax Eclipse XDB-C_8_柱作为第二维分析柱,该方法检出限为2.1~2.8 pg/mL,回收率>80%,重复性RSD<11.2%,已成功应用于食品样品的分析。Campone等^[62]^报告了一种快速自动分析牛奶和乳制品中AFM1的方法。该方法首先进行蛋白质沉淀和AFM1提取,然后采用在线SPE耦合UHPLC-MS/MS分析,实现AFM1的自动预富集和定量测定。对不同的乳制品进行了验证研究,方法定量限(1.5~2.4 ng/kg)约为欧盟法规No 1881/2006允许的人类直接食用的牛奶和乳制品中AFM1最高水平的1/25;加样回收率(86%~102%)和重复性(RSD<8%)符合欧盟法规No 401/2006规定的测定食品中真菌毒素水平的性能标准。此外,在研究的不同牛奶和乳制品中没有观察到基质效应。该方法测定精确度高于传统方法,同时优化了样品制备程序,减少了分析时间和成本,实现了高通量样品检测。

### 2.4 食品中其他化学危害物的检测

食品中危害物种类复杂繁多,除农药残留、兽药残留和生物毒素外,研究人员也将在线样品制备技术应用于其他污染物和残留物的测定,开发和建立了简单高效的检测方法。Cuthbertsona等^[65]^建立了在线SPE-LC-MS/MS定量测定饮用水中10种卤代苯醌类化合物的新方法,该方法定量限为0.2~166 ng/L,回收率为70%~111%, RSD<20%,满足实际检测需求,与离线方法相比,减少了浓缩步骤,节约样品、溶剂和标准品。Hou等^[66]^针对豆芽和绿豆芽中植物生长调节剂,建立了在线SPE-LC-MS/MS,并对在线净化条件进行优化,包括上样溶剂、上样溶液pH、洗脱溶剂、上样和洗脱流速等,该方法检出限低(2.34~20.2 ng/kg),回收率(86.0%~109%)和重复性(RSD≤9.8%)良好,可用于豆芽中植物生长调节剂的在线测定。

总体来看,对于食品中各类危害物的测定,与离线样品制备相比,在线样品制备分析时间和溶剂消耗大大减少,且灵敏度和准确度更好,表明在线样品制备技术可以应用于食品安全领域。

## 3 总结与展望

本文主要讨论了在线样品制备技术耦合液相色谱-质谱联用系统在食品检测领域的应用,详细介绍了常用在线样品制备技术(在线SPE、In-tube SPME和TFC)的原理和基本设备。3种在线样品制备技术原理和仪器具有相似性,均基于阀切换技术,将样品制备过程作为第一维度,通过六通阀(或十通阀)物理耦合到第二维度,实现在线自动化分析。本文对近十年来在线样品制备技术在兽药残留、农药残留、毒素污染以及其他危害物方面的应用进行了综述,发现基于在线样品制备技术所开发的方法样品制备过程简单,具有样品用量小、溶剂消耗少、样品通量高、分析成本低、结果准确、选择性强等优点,满足食品安全检测的要求。在线样品制备技术在食品安全检测领域发展迅速,今后可从以下几个方面进行更深入研究:(1)目前应用较为成熟的在线净化柱常以十八烷基键合硅胶或聚合物基质为填料,开发制备更多种类填料的在线净化柱,有助于扩大在线样品制备技术的应用范围;(2)与普通的检测器相比,高分辨质谱具有更好的精密度和准确度,尝试将在线样品制备技术与高分辨质谱仪串联起来,有利于在线样品制备技术方法的进一步发展;(3)针对不同食品基质进行评估对比,持续优化检测流程,提高检测效率。随着在线样品制备技术的进一步发展,特别是食品安全问题社会关注度不断提高,其在食品检测领域的应用会越来越广泛。
